# Inclisiran in a real-world single-center registry of patients with very high atherosclerotic cardiovascular risk

**DOI:** 10.1016/j.athplu.2025.09.001

**Published:** 2025-09-04

**Authors:** Antonio Centola, Michelangelo Carbonara, Serena De Nuzzo, Andrea Cuculo, Antonio Ruggiero, Giulio Campanale, Massimo Iacoviello, Paola Gargiulo, Pasquale Perrone Filardi, Natale Daniele Brunetti

**Affiliations:** a Department of Emergency and Urgency, Policlinico Riuniti University Hospital, Foggia, Italy; b Department of Medical & Surgical Sciences, University of Foggia, Foggia, Italy; c Department of Advanced Biomedical Sciences, University of Naples Federico II, Naples, Italy

**Keywords:** Inclisiran, LDL, Real-world, SIRNA, Registry

## Abstract

**Background:**

A mean relative 50 % reduction of LDL cholesterol (LDLc) levels was observed in randomized studies in patients treated with inclisiran, a small-interfering-RNA therapeutic agent that reduces hepatic synthesis of PCSK9. Less is known on real world and every-day practice patients.

**Methods:**

Fifty consecutive patients with or at risk of atherosclerotic cardiovascular disease (ASCVD) or familial hypercholesterolemia (FH) and treated with inclisiran according to Italian indications were enrolled in an observational study. LDLc levels were followed up.

**Results:**

26 patients had an acute coronary syndrome (ACS), 11 FH; 46 % were on high-intensity statin therapy, 68 % on combination therapy statin/ezetimibe.

Mean LDLc level of the study population was 118 ± 12 mg/dl at baseline, 80 ± 18 mg/dl after 3 months, and 70 ± 15 mg/dl after 6 months (ANOVA p < 0.001). The use of inclisiran was associated with significantly reduced LDLc levels of 21 % at 1 month and 44 % at 6 months.

LDLc reduction in patients with ACS was statistically significant and comparable with chronic CS. Patients receiving a background combination therapy (statin/ezetimibe) showed a greater reduction in circulating LDLc levels than patients using inclisiran alone. No significant side effects or treatment drop out were observed during follow up. Rates of subjects with LDLc levels below 70 mg/dl (Italian Drug Agency target) increased from 0 % at baseline to 56 % at 6 months (p < 0.001).

**Conclusions:**

In a real-world population 3–6 months of therapy with inclisiran provide consistent and effective reduction in LDLc levels without significant adverse side-effects.

## Highlights

• Real-world, single center population; 39 patients with ASCVD, 11 with FH.
• The use of inclisiran was associated with significantly reduced LDLc levels of 21% at 1 month and 44% at 6 months.
• LDL-c reduction in patients with ACS was statistically significant and comparable with CCS.
• Rates of subjects reaching LDL-c target levels according to level of CV risk (ESC guidelines) up to 74% (5±2 months FU).

## Graphical abstract

**Figure:**
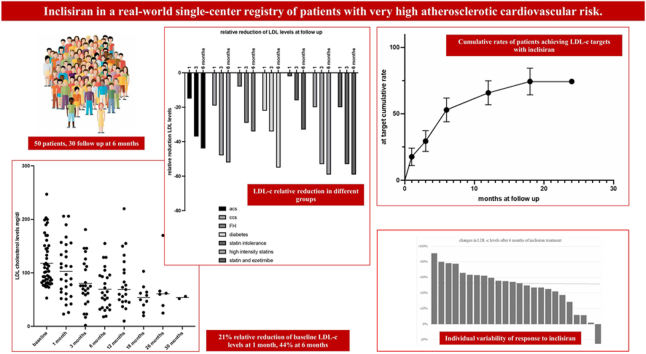

## 1 Introduction

Increased LDL-cholesterol (LDL-c) levels are considered as an etiologic factor in ischemic heart disease [1]. The reduction of LDL-c levels was associated to lower occurrence of cardiovascular (CV) events both in randomized controlled trials (RCT) and studies of Mendelian randomization, both in primary and secondary prevention [2]. In the last decades a multidrug LDL-c lowering therapy based on the use of inhibitors of hepatic synthesis of cholesterol, statins, intestinal absorption, ezetimibe, and monoclonal antibodies against PCSK9, PCSK9-inhibitors, combined according to the required reduction of LDL-c levels, was developed and implemented in the clinical practice and endorsed by international guidelines [3]. Lipid lowering therapy in combination of high intensity statins, ezetimibe, and PCSK9-inhibitors is able to reduce CV events by 20 % with a single mmol/l reduction of LDL-c levels [4].

Monoclonal inhibitors of PCSK9 showed their clinical efficacy in two pivotal RCTs, the FOURIER and the ODYSSEY-OUTCOMES studies [5]. More recently, a newer approach against PCSK9 has been adopted and shown as clinically effective in reducing LDL-c levels: inclisiran, a small interfering RNA (siRNA) therapeutic agent that reduces hepatic synthesis of PCSK9. In one trial, the LDL-c level was lowered by 53 % at 180 days after two doses of inclisiran [6]. Data from the same trial following the same patients over a period of 360 days suggested that inclisiran might provide sustained reductions in LDL cholesterol levels [7]. So, this new class of drug could reduce in the trials observation LDL-c levels of approximately 50 % only administered one subcutaneous injection every 6 months.

Despite a large body of evidence showing the clinical efficacy of inclisiran in reducing LDL-c levels coming from RCTs, less is known on inclisiran used in real-world and every-day practice patients [8]. We therefore sought to assess the efficacy of inclisiran in a single-center observational study.

## 2 Methods

This study is based on a real world, single-center cohort of patients with or at high risk of atherosclerotic CV disease (ASCVD) or familial hypercholesterolemia (FH, Dutch Score ≥8 points) and treated with inclisiran, enrolled from December 2022 to December 2023 in Italy, at Policlinico Riuniti University Hospital, Foggia.

Age, gender, type of dyslipidemia, CV diseases (acute coronary syndrome, ACS, coronary artery disease, CAD, peripheral artery disease, PAD), comorbidities, compliance to inclisiran therapy, prior therapy with statins and/or ezetimibe were recorded.

LDL-c, total cholesterol (TC), high-density lipoprotein cholesterol (HDL-C), and triglycerides (TG), were recorded for all patient at baseline (Time 0), after one month after the first dose of inclisiran (Time 1), after 3 months (Time 2), after 6 months (Time 3); data were also collected for a minority of patients even at 12, 18, 24 and 30 months as for a supplement of data analysis.

As indicated by ESC guidelines the LDL-c level goals were <55 mg/dl (<1.4 mmol/L) or <70 mg/dl (<1.8 mmol/L) according to the CV risk profile. In line with AIFA's (Agenzia Italiana del Farmaco) reimbursement indications, inclisiran is administered in primary prevention in patients, ≤80 years old, with heterozygous FH in case of detection of LDL >130 mg/dl despite six months of optimized medical therapy or statins intolerance; for secondary prevention can be used in patients ≤80 years old with heterozygous familiar or non-familiar hypercholesterolemia in case of LDL ≥70 mg/dl after 6 months of optimized medical therapy or after recent acute myocardial infarction (AMI).

### 2.1 Statistical analysis

Continuous variables were expressed as mean ± 95 % confidence intervals and compared with Student's t-test or Wilcoxon-Mann-Whitney as required, categorical variables as percentages and compared with χ^2^ test. Normality of distribution was assessed with Kolmogorov-Smirnov test. Repeated measures were analysed with ANOVA test for repeated measures or Kruskal-Wallis test with Dunn's correction for multiple comparison as required. A p < 0.05 was considered as statistically significant.

### 2.2 Sample sizing

On the base of prior data on LDL changes after inclisiran therapy and an expected change of at least 50 % over time, an 80 % power, an α of 0.05, and a drop-out of 1 in five patients, at least groups 10 subjects were required for the analysis.

## 3 Results

**Supplement Fig. S1:**
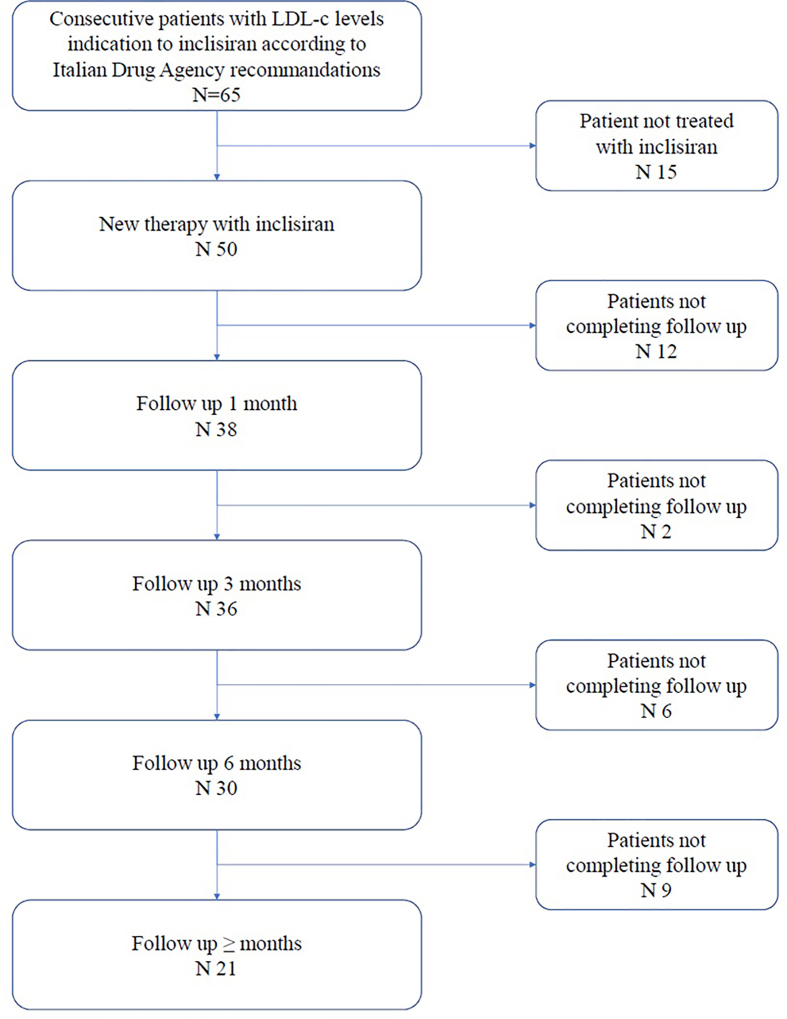
Patients enrolled in the study and follow up completion.

The clinical and laboratory characteristics of 50 consecutive patients enrolled in the study are given in Table 1, follow up completion is given in Supplement Fig. S1: 26 had an ACS (STEMI or NSTEMI), 11 FH, 22 non-acute CVD (chronic CS, PAD, or stroke), 14 were diabetic, 9 intolerant to statin therapy while 8 reported reduced tolerance to statins. Regarding lipid lowering therapy, 46 % were on high-intensity statin therapy, 26 % on moderate-intensity statin therapy, 68 % on combination therapy ezetimibe + statin, 10 % on monotherapy with statins or ezetimibe, 20 % were not taking any therapy.

**Table 1:** Clinical-laboratory characteristics of the population enrolled in the study.

Variable	Mean	95 %CI
male	72 %	
age	62	3
STEMI	34 %	
UA-NSTEMI	18 %	
CCS	20 %	
FH	22 %	
diabetes	28 %	
>10 years	64 %	
>3 cardiovascular risk factors	57 %	
	36 %	
PCI	68 %	
Multivessel coronary disease	20 %	
Peripheral artery disease	20 %	
stroke	4 %	
statin intolerance	18 %	
relative statin intolerance	16 %	

baseline		
total cholesterol	198	16
LDL	118	12
HDL	52	4
TG	157	26

1 month		
total cholesterol	174	21
LDL	103	19
relative reduction	−21 %	11 %
HDL	52	6
TG	118	21

3 months		
total cholesterol	150	23
LDL	80	18
relative reduction	−41 %	11 %
HDL	54	6
TG	115	25

6 months		
total cholesterol	138	21
LDL	70	15
relative reduction	−44 %	13 %
HDL	53	6
TG	121	28

ezetimibe	78 %	
rosuvastatin 5 mg	8 %	
10 mg	14 %	
20 mg	34 %	
atorvastatin 20 mg	2 %	
40 mg	8 %	
80 mg	4 %	

other statins	2 %	
high intensity statins	46 %	
statin + ezetimibe	42 %	

Legend. ST-elevation myocardial infarction, STEMI; unstable angina-non-ST-elevation myocardial infarction, UA-NSTEMI; chronic coronary syndrome, CCS; familial hypercholesterolemia. FH; percutaneous coronary intervention, PCI.

No inclisiran related side effect or therapy drop out was observed during study follow up.

**Fig. 1:**
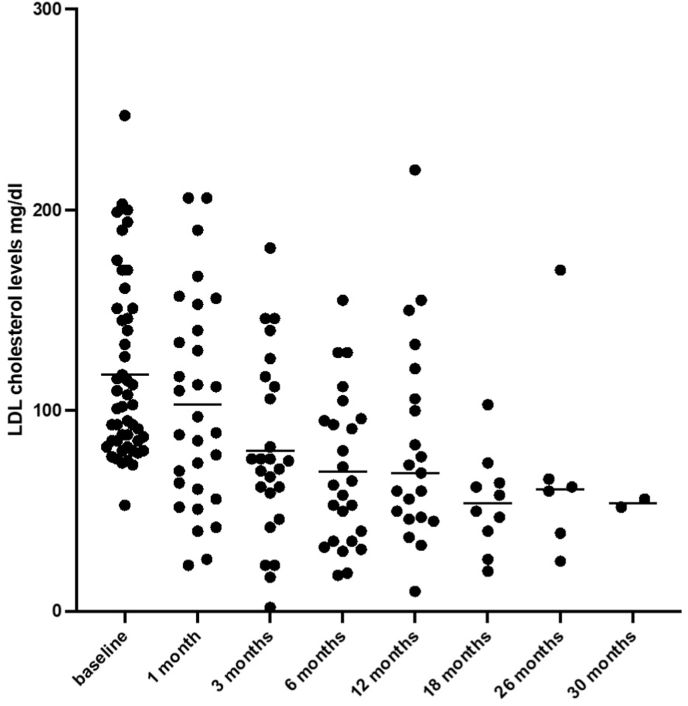
LDLc levels at baseline and follow up (p < 0.001).

**Supplement Fig. S2:**
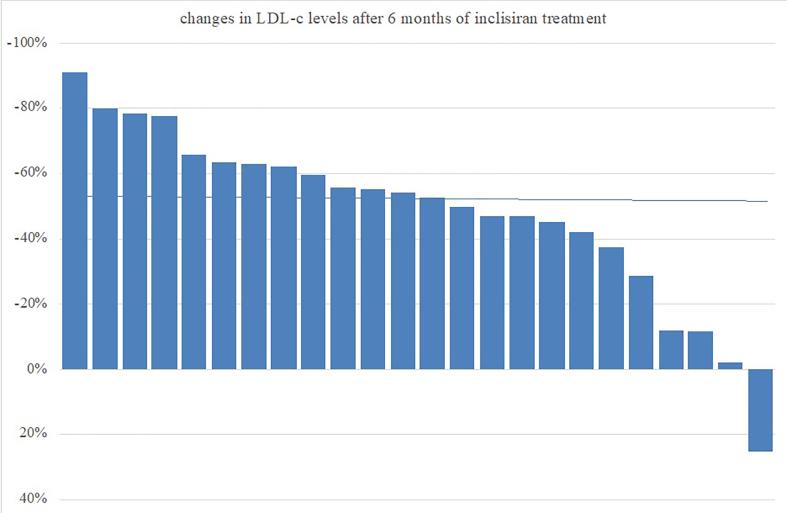
Individual variability of changes in LDL-c levels after 6 months of inclisiran therapy.

Mean LDL-c level of the study population was 118 ± 12 mg/dl at baseline, 103 ± 19 mg/dl after 1 month, 80 ± 18 mg/dl after 3 months, 70 ± 15 mg/dl after 6 months, 82 ± 21 mg/dl after 12 months, and 54 ± 15 mg/dl after 18 months (p < 0.001, Fig. 1). The use of inclisiran was associated with significantly reduced LDL-c levels of 21 % ± 11 % at 1 month, 41 % ± 11 % at 3 months, 44 % ± 13 % at 6 months. Individual variability of response is given in Supplement Fig. S2.

**Fig. 2:**
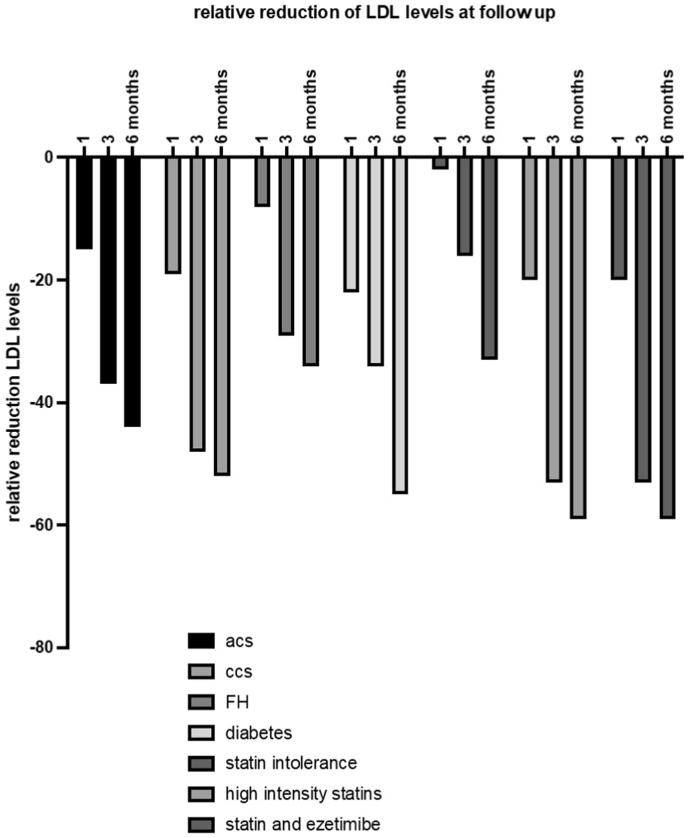
Relative reduction of LDLc levels at follow up across different sub groups.

LDL-c reduction in patients with ACS was statistically significant and comparable with CCS (−15 ± 13 % vs −19 ± 26 % at 1 month, −37 ± 12 % vs −48 ± 24 % at 3 months, −44 ± 18 % vs - 52 ± 32 % at 6 months, p ns in all cases) (Fig. 2, Table 2). The reduction in LDL-c levels in patients treated with high-intensity statins and inclisiran was greater than in patients using moderate-intensity statins or ezetimibe alone or no therapy. Patients receiving combination therapy (background statin + ezetimibe therapy) showed a greater reduction in circulating LDL-c levels compared to patients using inclisiran alone (−59 ± 5 % vs −33 ± 31 %); the sample, however, is underpowered to show statistically significant differences. Non-significant differences were observed comparing diabetic patients treated with inclisiran vs non-diabetics.

**Table 2:** Relative reduction of LDL-c levels at follow up across different sub groups.

ACS (vs CCS)	mean	95 %CI	mean	95 %CI	p
Relative reduction 1 month	−15 %	13 %	−19 %	26 %	0.7656
3 months	−37 %	12 %	−48 %	24 %	0.4216
6 months	−44 %	18 %	−52 %	32 %	0.6573

FH					
1 month	−8 %	30 %	−19 %	21 %	0.3479
3 months	−29 %	21 %	−44 %	15 %	0.2026
6 months	−34 %	39 %	−52 %	28 %	0.2689

Diabetes					
1 month	−22 %	13 %	−13 %	8 %	0.4630
3 months	−34 %	18 %	−41 %	10 %	0.6072
6 months	−55 %	17 %	−41 %	12 %	0.3940

statin intolerance					
1 month	−2 %	32 %	−20 %	20 %	0.1414
3 months	−16 %	21 %	−49 %	14 %	0.0049
6 months	−33 %	31 %	−55 %	26 %	0.1379

High intensity statin					
1 month	−20 %	10 %	−13 %	7 %	0.5162
3 months	−53 %	17 %	−31 %	13 %	0.0577
6 months	−59 %	5 %	−42 %	3 %	0.3081

statin or ezetimibe					
1 month	−10 %	20 %	−21 %	34 %	0.5619
3 months	−35 %	16 %	−20 %	26 %	0.3301
6 months	−41 %	27 %	−43 %	38 %	0.9252

statin and ezetimibe					
1 month	−20 %	10 %	−21 %	15 %	0.9367
3 months	−53 %	17 %	−20 %	25 %	0.0509
6 months	−59 %	5 %	−43 %	5 %	0.1026

Statin + ezetimibe vs or					
1 month	−20 %	10 %	−10 %	8 %	0.4089
3 months	−53 %	17 %	−35 %	15 %	0.1518
6 months	−59 %	5 %	−41 %	4 %	0.3962

**Fig. 3:**
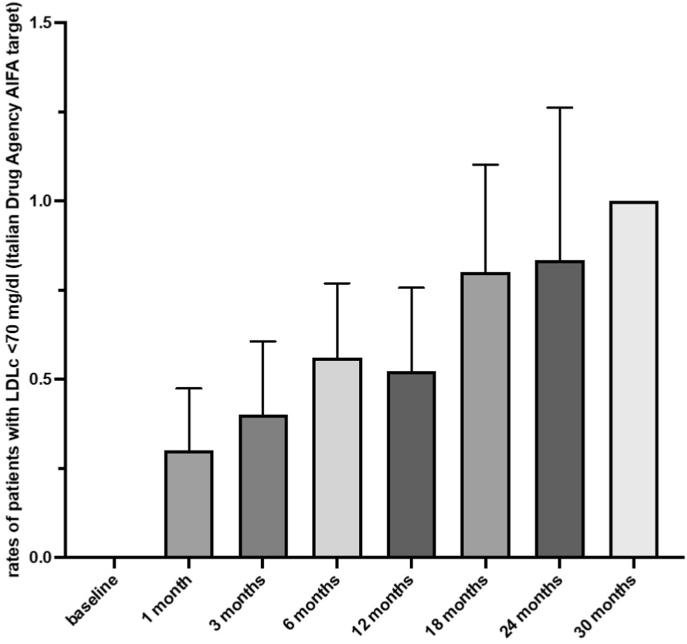
Rates of subjects with LDLc levels below 70 mg/dl, Italian Drug Agency target, after therapy with inclisiran (p < 0.001).

Rates of subjects reaching LDL-c AIFA target levels were 0 % at baseline, 30 % ± 17 % at 1 month, 40 % ± 20 at 3 months, 56 % ± 20 at 6 months, 83 % ± 33 % at 12 months (p < 0.001, Fig. 3).

**Fig. 4:**
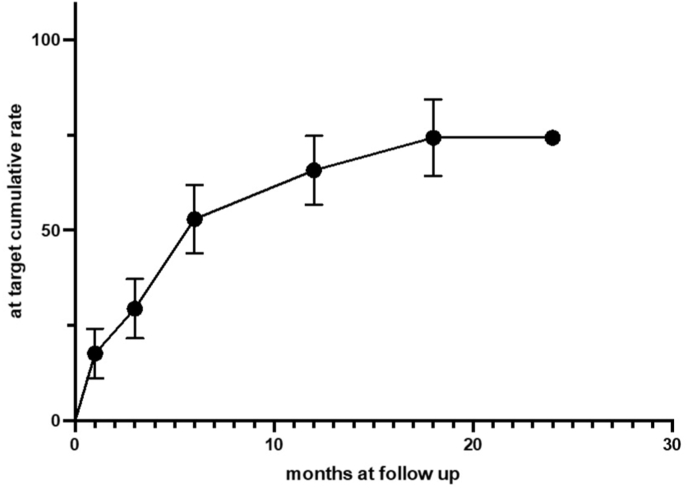
Cumulative rates of patients achieving LDL-c targets recommended by ESC guidelines.

Rates of subjects reaching LDL-c target levels according to level of cardiovascular risk as recommended by ESC guidelines (<55 mg/dl in all cases) accrued up to 74 % of subjects during a mean 5 ± 2 months follow up (p < 0.001, Fig. 4).

## 4 Discussion

In this observational study we showed that in a real-world population of patients with or at risk of ASCVD 3 months of therapy with inclisiran provide consistent and effective reduction in LDL-c levels without significant adverse side-effects. The relative reduction of LDL-c levels was consistent across sub-groups and also in patients with ACS. The larger relative reduction of LDL-c levels was observed in patients also in treatment with statins. We found in this real-world population a 15 % relative reduction of LDL-c after 1 month of therapy with inclisiran, of 39 % at 3 months, and of 46 % after 6 months. Mean cholesterol levels were 69 mg/dl after 6 months of therapy with inclisiran from 118 mg/dl at baseline.

Inclisiran was the first in class of LDL-c lowering SIRNA. In ORION 1 study, 501 patients were studied; after 180 days, a reduction in LDL-c was observed from 27.9 to 41.9 % after a single dose of inclisiran and from 35.5 to 52.6 % after two doses [9]. In the ORION-9 study, patients suffering from heterozygous FH, treated with a statin at the maximum tolerated dose, and treated with a statin at the maximum tolerated dose were enrolled; a reduction in LDL-c of 39.7 % was observed at day 510 [10]. The ORION-10 study enrolled patients with ASCVD, treated with high- and medium-intensity statins, who did not reach the target LDL-c <70 mg/dl; the study demonstrated that the therapeutic target was achieved in 84 % of patients, with a reduction of LDL-c by 52 % compared to placebo at day 510 [11]. The ORION-11 study enrolled patients with ASCVD or with equivalent ASCVD risk, treated with statins; in this RCT inclisiran was shown to significantly reduce the LDL-c value; in particular the average percentage change in LDL-c from baseline at day 510 compared to placebo was 50 %. At day 510, the LDL-c target of <1.8 mmol/L (70 mg/dl) was achieved by 82 % of ASCVD patients treated with inclisiran [12]. In patients with ASCVD risk equivalents, target LDL-c <2.6 mmol/L (100 mg/dl) was achieved by 78 % of patients treated with inclisiran [13].

RCTs patients, however, are well defined as for clinical characteristics, optimally treated and followed up; real world patients, by contrast, are often very different, more heterogeneous, characterized by less ideal follow up and adherence to therapy recommended by guidelines. Hence the need, in addition to RCTs, for registry studies able to evaluate the effectiveness of drugs in real populations and unselected patients. A registry study by the Italian Drug Agency, AIFA, published in 2022 evaluated the effectiveness of PCSK9i on an unselected population who had been prescribed these drugs; the data demonstrate that, in the real world, alirocumab and evolocumab are very effective in reducing LDL-c, but only 62 % of patients underwent a clinical re-evaluation of the ongoing therapy; adherence to therapy was very high, equal to 93 %. In another study by Gargiulo et al. adherence to monoclonal antibodies against PCSK9 therapy was near optimal as well [14].

Data on real world use of inclisiran have been recently published in a large population from Italy, 659 patients, showing a therapy persistence of 97 % and relative lipid lowering at 3 months of 51 % [15]. Our results show that inclisiran significantly reduced LDL-c levels by approximately 50 %, confirming the results of the ORION series studies.

Patients receiving statin/ezetimibe showed greater LDL-c reduction compared to those not on combination therapy, again in line with findings from larger Italian population [15]. Such results show an effect of inclisiran on a background of combination therapy with statins/ezetimibe different from that observed with alirocumab, achieving the same relative reduction of LDL levels either in association with high intensity statins or not [16]. Our findings are in line with other small registries on inclisiran [17].

In a retrospective analysis from Padam et al. on 80 patients who received a single dose of inclisiran, at 2 months after treatment initiation, mean baseline LDL-c fell from 3.5 ± 1.1 mmol/L by 48.6 % to 1.8 ± 1.0 mmol/L, with efficacy similar to that reported in RCTs [18]. In an observational multicenter registry including high cardiovascular risk patients from 12 lipid clinics in the Veneto region of Italy who started inclisiran, at 3 months of follow-up after inclisiran administration mean LDL-c levels significantly fell by 24.3 % and by 22.7 % at 9 months. LDL-c target achievement according to ESC/EAS 2019 guidelines was 64.7 % at 3 months and 62.3 % at 9 months. Patients on statin therapy had a greater LDL-c reduction compared to patients with statin intolerance. At 3 months greater LDL-c reduction was observed in patients with diabetes. In the multivariable analysis diabetes mellitus and background statin therapy were independent predictors for greater LDL-c reduction [19].

Our data are of particular interest because data at 1 month and at 12–24 months are also provided.

The individual response variability to inclisiran observed in this study was in line with other previous real world studies [20].

It is also noteworthy in our study the absence of therapy drop out and significant side effects in a small real-world populations of patients treated with inclisiran. Data from the AIFA OsMed Report on drug and lipid-lowering therapy in Italy demonstrate low levels of therapy persistence with statins [21], but much better persistence rates with PCSK9i [22].

We also found significant reduction of LDL-c levels after therapy with inclisiran in ACS patients, with relative rate reductions comparable with CCS patients. The relative reduction of LDL-c levels after inclisiran therapy was lower in our unselected patients than in the post-hoc sub-group analysis of pooled data from the ORION-10 and -11 studies [23], where patients were stratified into 3 groups according to the absence or presence of recent (>3 months < 1 year), non-recent (>1 year) acute myocardial infarction. However, the definition of recent acute myocardial infarction is different from those of ACS used to categorize patients in our population, so the difference in definition may be partly at least presumed as responsible for contrasting data. Our data are among the first on patients with recent ACS from a real-world dataset.

In general, however, observational real-world data from our small population are in line with RCTs and other observational data. Further studies on larger populations are warranted to confirm such preliminary data.

## 5 Conclusions

In a real-world population of patients with or at risk of ASCVD 3–6 months of therapy with inclisiran provide consistent and effective reduction in LDL-c levels without significant adverse side-effects. The relative reduction of LDL-c levels was consistent across sub-groups and also in patients with ACS. Larger relative reduction of LDL-c levels was observed in patients also in treatment with statins.

## Study limitations

This is an observational non-randomized study on a small single-centered population of patients with ASCVD or at high risk of ASCVD. Some LDL-c values are missing at intermediate follow up controls, thus justifying the apparent mismatch between number of patients completing follow up controls (Supplement Fig. S1) and values available at each follow up control (Fig. 1).

## Declaration of competing interest

The authors declare that they have no known competing financial interests or personal relationships that could have appeared to influence the work reported in this paper.
